# Modulation of the Caecal Gut Microbiota of Mice by Dietary Supplement Containing Resistant Starch: Impact Is Donor-Dependent

**DOI:** 10.3389/fmicb.2019.01234

**Published:** 2019-06-06

**Authors:** Claire Cherbuy, Déborah Bellet, Véronique Robert, Camille Mayeur, Andreas Schwiertz, Philippe Langella

**Affiliations:** ^1^Micalis Institute, INRA, AgroParisTech, Université Paris-Saclay, Jouy-en-Josas, France; ^2^MVZ Institut für Mikroökologie GmbH, Herborn, Germany

**Keywords:** resistant starch, gnotobiotic, human microbiota, butyrate, *Faecalibacterium prausnitzii*

## Abstract

Alterations in the gut microbiota have been associated with a wide range of pathologies and conditions. Maintaining a well-balanced microbiota is a key factor in sustaining good health. Our aim was to investigate the impact of a resistant starch-containing dietary supplement (SymbioIntest^®^) on the composition of the human gut microbiota and on intestinal short chain fatty acid (SCFA) concentration. Human microbiota-associated mice were used. Ex-germ-free mice were inoculated with fecal suspensions from four different donors. Three weeks later, the mice were orally gavaged for 1 month with either a daily dose of 10 mg of SymbioIntest^®^ or the vehicle (water) for the negative control group. The composition of the microbiota and SCFA levels were analyzed by 16S rRNA gene sequencing and gas chromatography, respectively. In three groups of mice, SymbioIntest^®^ supplementation increased the concentration of caecal butyrate. This was in conjunction with a remodeling of the gut microbiota. OTUs belonging to the *Bacteroidaceae*, *Porphyromonadaceae*, *Lachnospiraceae* and *Ruminococcaceae* families were affected. In two groups of mice the greatest changes in OTUs were seen in the *Faecalibacterium* genus. The supplementation’s highest impact was observed in mice inoculated with gut microbiota containing a lower number of *Ruminococcaceae* and *Faecalibacterium* and a higher number of *Prevotellaceae*. SymbioIntest^®^ supplementation elicited a beneficial effect on the healthy adult gut microbiota by increasing caecal butyrate production and health-promoting taxa. We highlight the fact that screening the gut microbiota may be used for predicting individualized responses to dietary interventions and thus developing personalized nutritional strategies.

## Introduction

Human beings and microbes co-exist in symbiosis. Most of the body’s microbes are found in the distal part of the digestive tract where colonization level is as high as 10^11^ microbes/g of content. The human gut microbiota is dominated by two major phyla: the *Bacteroidetes* and the *Firmicutes*, followed by subdominant phyla such as *Actinobacteria* and *Proteobacteria* (Qin et al., 2010; Human Microbiome Project Consortium, 2012). The interactions between the host and the gut microbiota affect both the health and well-being of the host as they impact several aspects of host physiology including organ development, maturation of the immune system and energy metabolism (Sommer and Backhed, 2013). Today there is a growing body of evidence that links irregular microbiota-host interactions to an increasing number of modern multi-factorial immune-mediated and metabolic pathologies such as obesity, type 2 diabetes, inflammatory bowel disease, allergies and asthma (Sommer and Backhed, 2013; Petersen and Round, 2014).

Diet greatly affects the gut microbiota and its interactions with the host (Goldsmith and Sartor, 2014). Carbohydrates are the principal carbon and energy source for the gut microbiota, that produce one of the large panel of enzymes involved in the hydrolysis of wide range of complex polysaccharides (Gill et al., 2006; Tasse et al., 2010). Starch is a dietary carbohydrate that is a popular nutritional source for both humans and animals. Besides digestible starch, which is rapidly or slowly hydrolysed, a variable fraction, called resistant starch (RS), resists digestion in the small intestine and is fermented in the large intestine, where it provides nutrients for the gut microbiota. Many studies highlight that RS might contribute to positive health outcomes and this could involve a beneficial effect on the function of the large bowel (Bindels et al., 2015). In particular, RS is fermented by the gut microbiota into short chain fatty acids (SCFA) (Shen et al., 2017) that have a diverse range of physiological effects on the host (Macfarlane and Macfarlane, 2012). This is concomitant to a shift in microbiota composition toward the increase of butyrate-producing bacteria in humans (Walker et al., 2011) or in pigs (Haenen et al., 2013).

Our objective was to further investigate the impact of an RS-containing dietary supplement, SymbioIntest^®^ on the composition of the human gut microbiota and intestinal SCFA concentration. To limit the human dietary intervention variables (host genotype, diet, environmental conditions), we used ex-germfree mice colonized by microbiota coming from healthy volunteers. As it has been shown that there is a strong inter-individual difference in response of each microbiota’s membership and function to a dietary ingredient (Walker et al., 2011; Smits et al., 2016), we used four distinct human-derived gut microbial communities to test how different microbiota respond to a defined change in SymbioIntest^®^ supplementation. We highlight the fact that individual gut microbiota respond in a variable manner to dietary interventions. This suggests that screening the gut microbiota may be used for predicting individualized responses to dietary interventions and thus developing personalized nutritional strategies.

## Materials and Methods

### Animal Experimentation, Donors, and SymbioIntest^®^ Product

Experiments were performed on the Anaxem platform of the MICALIS Institute (INRA, Jouy-en-Josas, France). The Anaxem facilities are accredited by the French “Direction Départementale de la Protection des Populations (DDPP78),” accreditation number A78-322-6. All procedures involving animal experimentation were carried out according to the European guidelines for the care and use of laboratory animals under the authority of a license issued by the French Veterinary Services (authorization number 78–122 specific to CC) and were approved by the French “Ministère de l’Enseignement Supérieur et de la Recherche” (authorization number APAFIS#3441-2016010614307552).

Ethical considerations are in line with the French guidelines on fecal sample collection for research use. Healthy donors signed informed consent forms for the use of their fecal material in this study together with an agreement of confidentiality. All forms signed by the donors were previously validated by INRA. Characteristics of the donors are given in Table 1. None of the donors took antibiotics in the 2 months preceding the stool collection. SymbioIntest^®^ was provided by Symbiopharm (Herborn, Germany). It contains RS-type 3 and its composition can be found in https://www.symbiopharm.de/en/products/symbiointest.html.

**TABLE 1:** Donor characteristics.

**Donor**	**Sex**	**Age**	**BMI**	**Diet**
D1	F	60 < age > 70	20 < BMI > 25	Omnivorous/diversified
D2	M	30 < age > 40	25 < BMI > 30	Omnivorous/diversified
D3	F	60 < age > 70	20 < BMI > 25	Omnivorous/diversified
D6	M	30 < age > 40	25 < BMI > 30	Omnivorous/diversified Regular consumption of vegetables

*Characteristics of the 4 healthy donors whose fecal samples were used in the study. F, female; M, male volunteers consuming omnivorous diets; BMI, body mass index. The donors were asked to briefly describe their diet.*

### Procedure Concerning the Human Microbiota-Associated (HMA) Mice

All GF mice (7 to 8 week-old males, C57Bl/6) were purchased from the GF rodent breeding facilities of the CNRS-TAAM (transgenesis, archiving and animal models) center (Orléans, France). After receipt, GF mice were left undisturbed for 8 days before starting the experiment. Mice were kept in cages (4–5 mice/cage) whose dimensions were 29 cm long, 18 cm wide, and 15 cm high. The bedding was sterile wood shavings. Mice were given free access to autoclaved tap water and a γ-irradiated (45 kGy) standard diet (R03; Scientific Animal Food and Engineering, Augy, France).

A diagram of the experimental flow is given in Supplementary Figure 1. The freshly passed feces of four healthy donors were used to obtain human microbiota-associated (HMA)-mice. Each group of mice was randomly inoculated with the fecal sample coming from one of the four donors-D1, D2, D3, and D6. Fecal samples were immediately placed into an anaerobic chamber and diluted (10^–2^) in Brain Heart Infusion medium. Then the inocula were immediately transferred into the isolator to perform oral gavage. The mice were each inoculated with 100 μl of fecal suspension. One tube of inoculum was used for each pair of GF mice to limit the exposure of EOS strains to ambient air. Three weeks after the fecal transplant, the feces of the inoculated mice were collected to analyze the composition of the transferred microbiota.

### Supplementation Design and Sampling

Supplementation with SymbioIntest^®^ was initiated 3 weeks after the transfer of the human gut microbiota and given daily by oral gavage over a period of 4 weeks. The daily dose of SymbioIntest^®^ given to mice was 10 mg. It was diluted in water. Given the weight ratio between humans and mice, this is close to the amount of SymbioIntest^®^ recommended for human consumption (i.e., 10 g corresponding to 5 g of RS3). Control mice were given the same volume of water per day by oral gavage. At the end of the four-week supplementation period, the mice were killed by cervical dislocation and the caecal contents were removed. The samples were immediately weighed and stored frozen at −80°C until DNA extraction and SCFA measurements were performed. The weight of the mice was similar between the different groups at the end of the experiment (Supplementary Figure 2).

### SCFA Analysis of Caecal Samples

SCFA (acetate, propionate, and butyrate) content was determined by gas chromatography (Nelson 1020, Perkin-Elmer, St Quentin en Yvelines, France). The samples were extracted with water (wt g/vol), centrifuged at 17,000 × *g* for 10 min, and the supernatant collected. The proteins were precipitated using a phosphotungstic acid saturated solution. A volume of 0.1 mL of the supernatant was analyzed using a gas–liquid chromatograph (Autosystem XL; Perkin Elmer, Saint-Quentin-en-Yvelines, France). All samples were analyzed in duplicate. The data was collected and peaks integrated using Turbochromv6 software (Perkin Elmer, Courtaboeuf, France).

### Analysis of the Caecal Microbiota Community by 16S rRNA Gene Survey Analysis

Total bacterial DNA was extracted from the collected samples according to the protocol described in Godon et al. (1997). DNA concentration and integrity were determined spectrophotometrically using a NanoDrop instrument and visually by electrophoresis on a 1% agarose gel containing ethidium bromide. The DNA concentration values were between 0.6 and 1 μg/μl. The size distribution of the DNA extracted from the fecal and caecal samples estimated by agarose gel electrophoresis showed that most of the DNA was high molecular weight (>20 kb) with no significant shearing. Taken together, these observations suggest that the extracted DNA was of good quality, suitable for downstream processing.

The V3-V4 hyper-variable region of the 16S rRNA gene was amplified with the primers F343 (CTTTCCCT ACACGACGCTCTTCCGATCTTACGGRAGGCAGCAG) and R784 (GGAGTTCAGACGTGTGCTCTTCCGATCTTACCAGG GTATCTAATCCT). The PCR reactions were performed using 10 ng of caecal DNA, 0.5 μM primers, 0.2 mM dNTP, and 0.5 U of the DNA-free Taq-polymerase, MolTaq 16S DNA Polymerase (Molzym). The amplifications were carried out using the following profile: 1 cycle at 94°C for 60 s, followed by 30 cycles at 94°C for 60 s, 65°C for 60 s, 72°C for 60 s, and finishing with a step at 72°C for 10 min. The PCR reactions were sent to the GeT-PlaGe platform (INRA, Toulouse) for sequencing using Illumina MiSeq technology. Single multiplexing was performed using home made 6 bp index, which were added to R784 during a second PCR with 12 cycles using forward primer (AATGATACGGCGACCACCGAGATCTACACTCTTTCCCTA CACGAC) and reverse primer (CAAGCAGAAGACGGC ATACGAGAT-index-GTGACTGGAGTTCAGACGTGT). The resulting PCR products were purified and loaded onto the Illumina MiSeq cartridge according to the manufacturer’s instructions. The quality of the run was checked internally using PhiX, and then each pair-end sequence was assigned to its sample with the help of the previously integrated index. Each pair-end sequence was assembled using Flash software (Magoc and Salzberg, 2011) using at least a 10 bp-overlap between the forward and reverse sequences, allowing 10% of mismatch. The lack of contamination was checked with a negative control during the PCR (water as template). The quality of the stitching procedure was checked using four bacterial samples that are run routinely in the sequencing facility in parallel to the current samples.

### 16S rDNA Gene Sequences and Statistical Analysis

Sequences were first analyzed using the FROGS pipeline to obtain the OTU (Operational Taxonomic Units or phylotypes) abundance table. The successive steps involved de-noising and clustering of the sequences into OTUs using SWARM; chimera removal using VSEARCH; taxonomic affiliation for each OTU using both RDP Classifier and NCBI Blast+ on Silva SSU 119 and 123 (Escudie et al., 2017). Statistical analyses were performed using “R” language and environment version 3.2.3. β-diversity (UniFrac and weighted UniFrac dissimilarity), α-diversity measurements and analysis of the differences in OTUs between samples were performed using the add-on package “Phyloseq” (McMurdie and Holmes, 2013). Differences in the microbial communities between control and treated groups were evaluated using constrained analysis of principal coordinates and permutational multivariate ANOVA. Statistical differences between control and treated groups for individual OTU abundance and SCFA concentrations were calculated using the Mann-Whitney test with Benjamini-Hochberg false discovery rate correction. Statistical significance was set at *P* < 0.05.

## Results

### Analysis of the Four Donors’ Gut Microbiota

**FIGURE 1:**
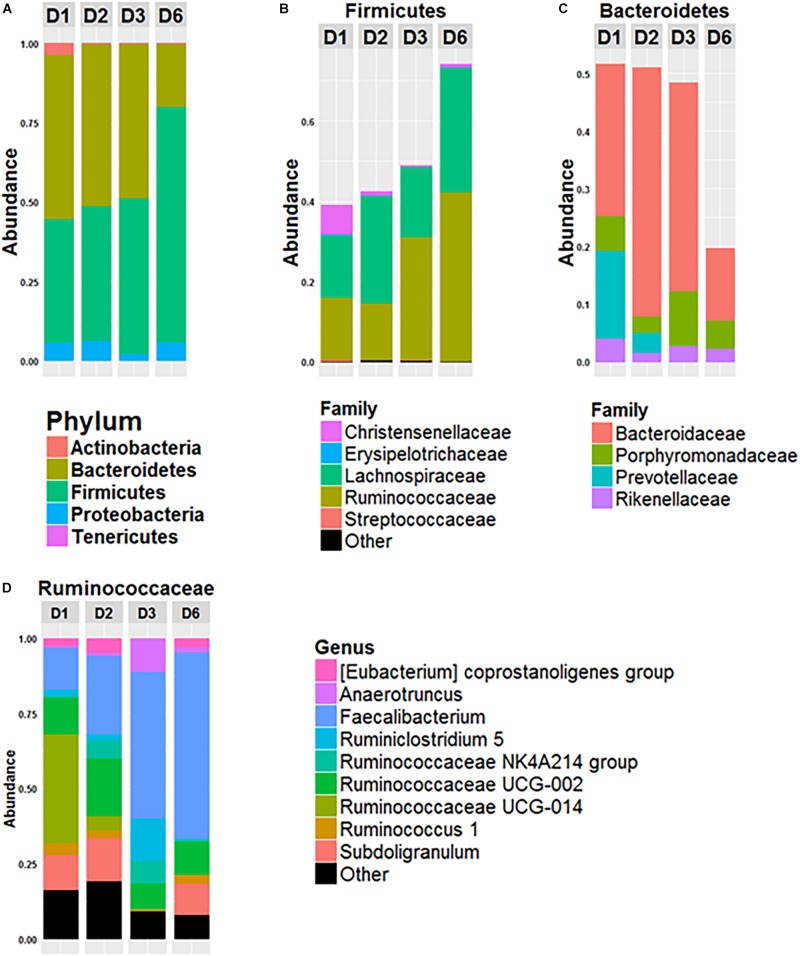
Composition of fecal microbiota of the 4 donors. Relative abundance of phyla **(A)**, of bacterial families of *Firmicutes* **(B)** and of *Bacteroidetes* **(C)** and relative abundance of bacterial genus within the *Ruminococcacae* Family **(D)** in the fecal microbiota of donor 1 (D1), donor 2 (D2), donor 3 (D3), and donor 6 (D6). Data is based on the sequences of the 16S rRNA gene.

The composition of the gut microbiota of the four donors (D1, D2, D3, and D6) was analyzed (Figure 1). The gut microbiota of D6 differed from the others at the phylum level with a higher *Firmicutes/Bacteroidetes* ratio (*Firmicutes* represented 74% of the total OTUs and *Bacteroidetes* 19%). In contrast, these two major phyla were more equally represented in the microbiota of the other three donors, with *Firmicutes* representing 51, 51, and 49% and *Bacteroidetes* representing 39, 42 and 48% of all OTUs for D1, D2, and D3, respectively (Figure 1A). *Lachnospiraceae* and *Ruminococcaceae* made up the two major families of *Firmicutes* in each donor, but they were present in different amounts (Figure 1B): for D1, *Ruminococcaceae* and *Lachnospiraceae* each represented 15% of all OTUs; for D2 *Lachnospiraceae* was higher than *Ruminococcaceae*: 26 and 14%, respectively. In D3 and D6, *Ruminococcaceae* was the most abundant of all OTUs, 30 and 42%, respectively, whereas *Lachnospiraceae* represented 17 and 30%. For the phylum *Bacteroidetes* (Figure 1C), *Bacteroidaceae* was predominant in all donors: *Bacteroidetes* represented 26, 42, and 36% of the all OTUs for D1, D2, and D3, respectively and 12% for D6. *Prevotellaceae* was present in the gut microbiota of D1 and D2 (15 and 3% of all OTUs for D1 and D2) but was not detected in D3 or D6 (Figure 1B). As shown in Figure 1D, representation of the *Ruminococcaceae* family was heterogeneous among the donors. In D3 and D6, the dominant genus of *Ruminococcaceae* was *Faecalibacterium* which represented 48% and 62% of *Ruminococcaceae*, respectively, whereas *Faecalibacterium* represented 13 and 26% of *Ruminococcaceae* for D1 and D2.

### Implantation of Human Microbiota in Recipient GF Mice

**FIGURE 2:**
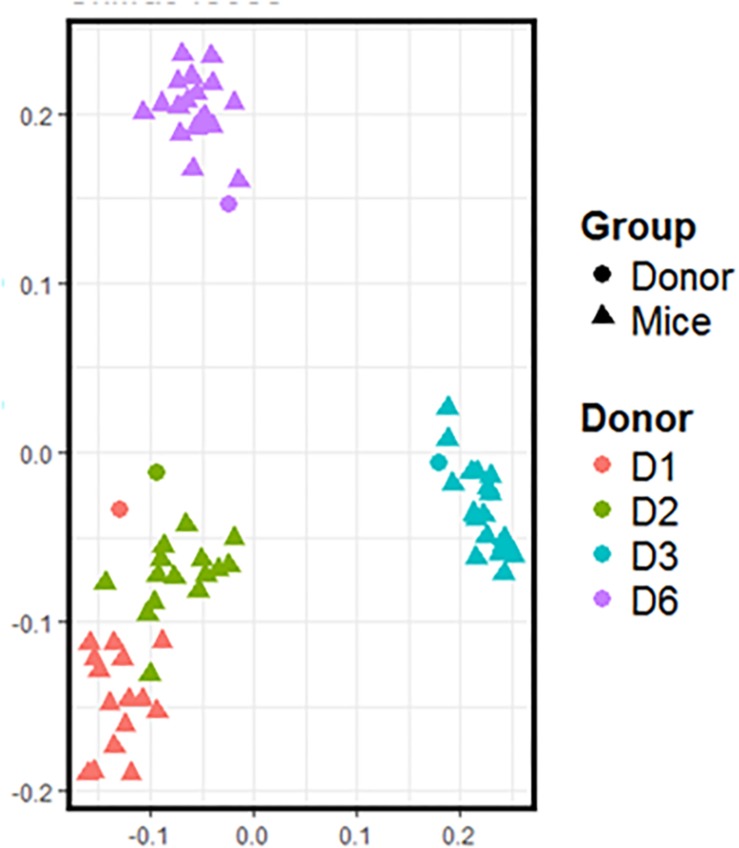
16*S* rRNA gene surveys (analyzed by unweighted UniFrac-based PCoA) from the fecal microbiota of inoculated mice and those of the respective donor. Each dot represents one individual (donor or mouse).

Freshly obtained stools from D1, D2, D3, and D6 were used to inoculate recipient GF mice. Following the transfer, the mice were left undisturbed to allow stabilization of the microbiota. Three weeks after the transfer, the day before the start of supplementation, we recovered the feces of inoculated mice to compare their composition with that of the donor (Figure 2). Data shows that the microbiota from inoculated mice cluster with the gut microbiota of the respective donor. Consistent with Figure 1, the gut microbiota of mice inoculated with fecal samples coming from D1 and D2 are closer than that of mice inoculated with fecal samples coming from D3 and D6. The gut microbiota of these two groups of mice also differ from each other.

### Effect of Supplementation With SymbioIntest^®^ on the Whole Gut Microbiota Community

Three weeks after the transfer of donor microbiota, the mice were split into two groups, one supplemented daily with 10 mg of SymbioIntest^®^ and the other with water as a negative control. Four weeks after the start of supplementation, the mice were killed and the caecal contents were recovered to determine the impact of supplementation on the gut microbiota structure.

**FIGURE 3:**
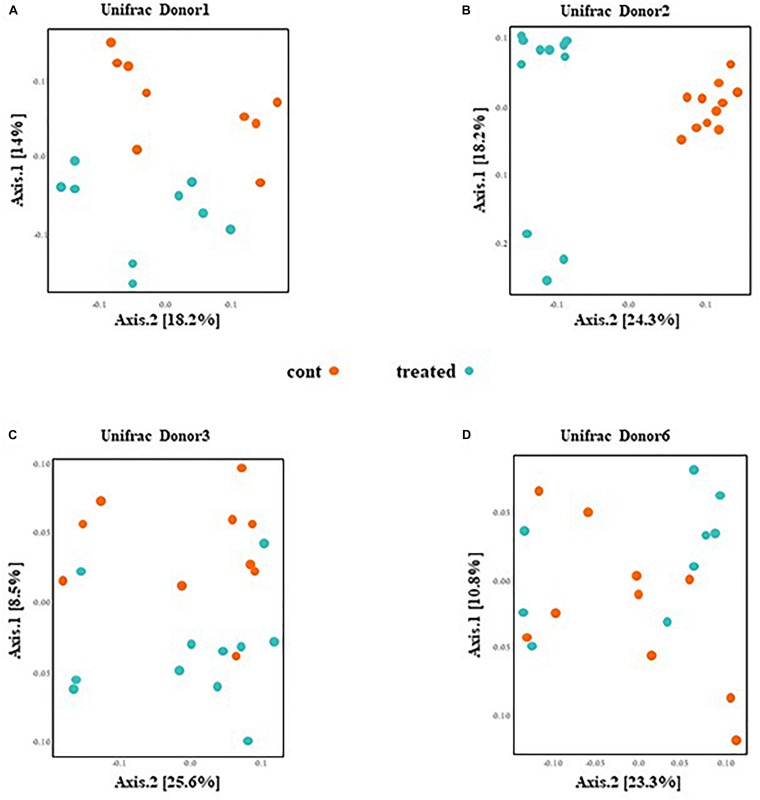
Caecal contents were collected from mice inoculated with feces from donor D1 **(A)**, donor D2 **(B)**, donor D3 **(C)**, and donor D6 **(D)** after 4 weeks of daily supplementation with SymbioIntest^®^ or water. 16S rDNA gene sequences were obtained and the composition analyzed by Unifrac distance. Cont and treated: data obtained from water and SymbioIntest^®^-supplemented mice, respectively. Each dot represents one mouse with 9 to 11 mice analyzed per groups.

PCoA presented in Figure 3 are based on UniFrac distance metrics. This data shows that the gut microbiota of mice inoculated with fecal samples of D1 and D2 cluster according to the treatment. Therefore, this reveals distinct differences in bacterial communities between control mice and mice supplemented with SymbioIntest^®^ for these groups (Figures 3A,B). This was confirmed with further statistical analysis (Constrained analysis of the principal coordinates and permutational multivariate ANOVA) that shows a significant shift in community composition between control and treated groups (*p* < 0.05 for D1 and D2). According to these tests, supplementation with SymbioIntest^®^ was responsible for 20% and 26% of the difference in bacterial communities for mice inoculated with the gut microbiota of D1 and D2, respectively. In contrast, PCoA reveals no distinct clustering between the gut microbial communities of mice inoculated with the microbiota of D3 or D6, whether the mice received supplementation or not (Figures 3C,D). Accordingly, constrained analysis of principal coordinates and permutational multivariate ANOVA found no significant statistical differences between the control and the treated groups in mice inoculated with the gut microbiota of D3 or D6. Thus, our data shows that the response of the gut microbiota to supplementation differed depending on the initial human gut microbiota used to inoculate the mice. Indeed, the global structure of the gut microbiotas of D1 and D2 (Figures 3A,B) was modified by supplementation but not that of the D3 or D6 gut microbiota (Figures 3C,D).

Other parameters of microbial communities were unchanged (α-diversity; data not shown), as well as weighted UniFrac analyses (Supplementary Figure 3). This suggests that differences between samples appear to be driven by substantial changes in the less abundant taxa, whereas abundant OTUs seem to be less affected by the supplementation.

### SymbioIntest^®^ Remodels *Bacteroidaceae*, *Lachnospiraceae*, *Porphyromonadaceae*, and *Ruminococcaceae*

**FIGURE 4:**
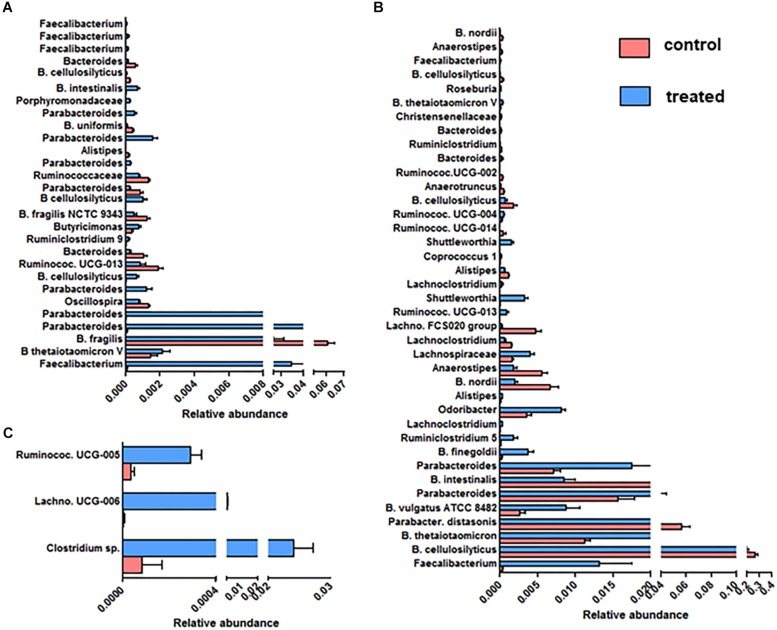
OTUs significantly modified between the control and SymbioIntest^®^ -treated groups in mice inoculated with the gut microbiota of D1 **(A)**, D2 **(B)**, D3 **(C)**. Results are based on the sequences of rRNA 16S. Data is given as mean ± SEM of the relative abundance of each OTU in the control and treated group. *n* = 9 to 11 mice per groups. Information on the Family, Genus or Species (if available) is given.

We further focused on the number and identity of OTUs affected by the supplementation with SymbioIntest^®^ (Figure 4). We found that 28, 39 and 3 OTUs were significantly modified between the control and treated group, respectively, in mice inoculated with the fecal microbiota of D1 (Figure 4A), D2 (Figure 4B) and D3 (Figure 4C). We found no modified OTUs in the group of mice inoculated with D6. Overall, we found that the OTUs belonging to the bacterial families *Bacteroidaceae*, *Porphyromonadaceae*, *Lachnospiraceae*, and *Ruminococcaceae* were modified by supplementation with SymbioIntest^®^ but in a donor-specific manner. In both groups of mice inoculated by the fecal microbiota of D1 and D2 we found a significant increase of OTUs corresponding to *Faecalibacterium*, that is the genus of the *Ruminococcaceae* family mostly impacted by SymbioIntest^®^ supplementation in these groups (Figures 4A,B). For D1, two other OTUs, corresponding to *Butyricimonas* and *Ruminiclostridium 9*, both butyrate producers, increased after supplementation but their level remained low compared to *Faecalibacterium*. In the group of mice inoculated with the fecal sample of D2, in addition to the rise in *Faecalibacterium*, we found that members of the genus *Ruminiclostridium 5*, *Lachnoclostridium*, *Ruminococcaceae UCG-013*, *Shuttleworthia*, *Ruminococcaceae UCG-004*, and *Roseburia* also increased (Figure 4B). In addition to an increase in these members, we observed a remodeling in the families of *Lachnospiraceae* and *Ruminococcaceae*. In fact we observed a decrease in the level of OTUs belonging to *Ruminococcaceae* UCG-013, *Ruminococcaceae* and *Oscillospira* in mice inoculated with the fecal samples of D1 (Figure 4A). This remodeling was more marked in mice inoculated with the fecal samples of D2 and we found a greater number of OTUs belonging to *Lachnospiraceae FCS020*, *Lachnoclostridium*, *Anaerostipes*, *Ruminiclostridium*, *Ruminococcaceae UCG-002*, *Ruminococcaceae UCG-014*, *Anaerotruncus* that decreased after supplementation.

The abundance of many clusters belonging to the *Porphyromonadaceae* family, mainly of the *Parabacteroides* genus, increased after supplementation (Figures 4A,B). Nevertheless, we found that an abundant OTU corresponding to *Parabacteroides distasonis* decreased after supplementation in mice inoculated with the fecal sample of D2 (Figure 4B).

The *Bacteroides* OTUs were also modified following supplementation, either positively or negatively. In our study, *B. thetaiotaomicron*, one of the most abundant member of the *Bacteroides* genus, which is able to degrade diverse complex polysaccharides, increased in two groups of mice that received SymbioIntest^®^ (Figures 4A,B). The impact of supplementation on *B. cellulosilyticus*, a species which is a cellulolytic *Bacteroides*, was OTU-dependent. Some OTUs corresponding to *B. cellulosilyticus* were positively affected in mice inoculated with the fecal sample of D1, whereas others decreased, whether the mice were inoculated with the fecal samples of D1 or D2. *Bacteroides fragilis* was also less abundant in supplement-fed mice that were inoculated with the feces of D1 (Figure 4A). In mice inoculated with the feces of D2, OTUs corresponding to *B. vulgatus* and *B. finegoldii* increased.

We found that 3 OTUs in mice inoculated with the gut microbiota of D3 were significantly increased by the supplementation (Figure 4C) – one belonging to the *Lachnospiraceae* family; the others belonging to *Ruminococcaceae*. No increase of OTUs corresponding to *Faecalibacterium* was observed in this group.

### SCFA Analysis

**FIGURE 5:**
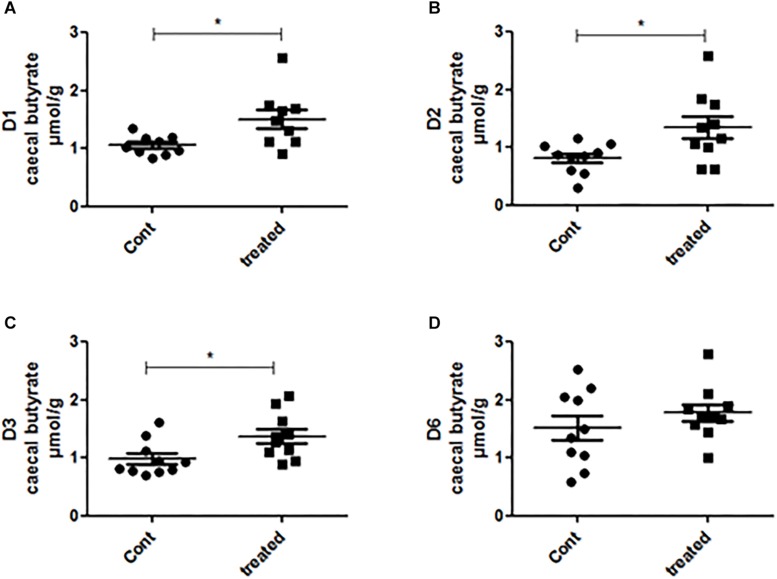
Caecal contents were collected from mice inoculated with feces from donors D1 **(A)**, D2 **(B)**, D3 **(C)**, and D6 **(D)** after 4 weeks of daily supplementation with SymbioIntest^®^ and processed for butyrate measurement as described in the methods section. Cont and treated: caecal contents obtained from control and SymbioIntest^®^-supplemented mice, respectively. Each dot represents one mouse with 9 to 10 mice analyzed per groups. ^*^p < 0.05.

We further measured the acetate, propionate and butyrate concentrations in the caecal samples of the control and treated group. We found that the treatment did not alter the caecal acetate concentration of any mice (Supplementary Figure 4). The propionate concentration was higher in the supplement-fed mice than in the control mice for two groups of mice: those that had been inoculated with the gut microbiota of D2 and D3 (Supplementary Figure 5). The caecal butyrate concentration was significantly higher in mice supplemented with SymbioIntest^®^ than in control mice for those inoculated with the gut microbiota of D1, D2, and D3, but not D6 (Figure 5). However, in contrast to what was found at the caecal level, when we measured the butyrate concentration in the fecal samples, we did not find any difference between the supplement-treated and control group mice, irrespective of the donor used (data not shown). This can be explained by the fact that 95% of the SCFAs produced are rapidly absorbed by colonocytes while the remaining 5% are secreted in the feces, canceling out the differences observed in the caecum. We subsequently measured lipocalin2 (lcn2) levels in the feces of all the different groups of mice (Supplementary Figure 8). Our findings show that there is no difference between the control and the prebiotic-treated groups irrespective of the donor used to inoculate the mice. This means that in a non-inflammatory situation, the prebiotic treatment has no impact on levels of lcn2.

## Discussion

We have shown that SymbioIntest^®^ supplementation leads to a significant increase in caecal butyrate concentrations in three groups of mice inoculated with the gut microbiota of three different donors. This is accompanied by a remodeling of the gut microbiota where OTUs are positively or negatively impacted. The increase in caecal butyrate concentration can involve different members of the gut microbiota among the groups of responsive mice.

The rise in *Faecalibacterium* OTUs is likely one of the main reasons for the increase in butyrate production in mice inoculated with D1 and D2 fecal microbiota. In the group of mice inoculated with the D1 fecal samples, the increase in caecal butyrate seems to be even quite exclusive to the increase in *Faecalibacterium*. In the group of mice inoculated with the D2 fecal sample, in addition to the increase in *Faecalibacterium*, there is a more diverse landscape of butyrate-producing bacteria post-supplementation that could also explain the increase in caecal butyrate concentration. In the group of mice inoculated with the D3 fecal sample, the OTUs modified by the supplement were all butyrate-producing bacteria but we observed no increase in *Faecalibacterium*.

Supplementation was seen to have the greatest effect on mice inoculated with gut microbiota having a lower abundance of *Ruminococcaceae*, with a large difference in *Faecalibacterium* and a higher abundance of *Prevotellaceae*. The group of mice resistant to SymbioIntest^®^ supplementation had a high level of *Ruminococcaceae* and *Faecalibacterium* and a low abundance of *Prevotellaceae*. In this case we suggest that the *Ruminococcaceae* and *Faecalibacterium* families were already saturated with no further impact possible on these bacterial groups.

As only 15% of gut bacterial lineages are shared between humans and mice (Ley et al., 2005; Nguyen et al., 2015), we conducted our study on HMA mice, thus enabling us to analyze gut microbiota members found in humans. In our study, the transferred gut microbiota clustered with that of the respective donors 3 weeks after the inoculation. This revealed that a stable human gut community, similar to that of the donor, became established in the recipient mice. This is consistent with previous studies where it was found that 85% of human microbiota genus-level taxa can be successfully transferred to germ-free mice (Turnbaugh et al., 2009).

In line with previous data, obtained from a similar model (Smits et al., 2016), our study shows that, using HMA mice, there were inter-individual responses to the dietary supplement depending on each subject’s initial microbiota make-up. In other words, the impact of SymbioIntest^®^ was not the same across the board and differed according to the composition of the initial donor’s microbiota. This marked inter-individual response to RS has been previously observed in humans (Maier et al., 2017). However, as we used 4 donors in this study, this variable response to the supplement could be confirmed by using a larger number of donors. Interestingly, we can notice that the gut microbiota of D6 (the donor claiming a regular consumption of vegetables) is the least responsive to supplementation. This could suggest that this gut microbiota was already adapted for complex polysaccharide breakdown and additional intake of prebiotic has little or no effect.

In the study of Walker et al. (2011), it was reported that the gut microbiota of subjects who have a low RS3 fermentation exhibit a lower number of *Ruminococcus bromii* than subjects who have a high capacity for RS3 breakdown. Therefore, variation in the level of *R. bromii* and its close relatives might be one of the primary causes of variable fermentation capacity of this component of the diet (Walker et al., 2011). In our study all the gut microbiota of the donors exhibited similar and high levels of *R. bromii* (Supplementary Figure 6), making all potentially able to breakdown RS3. Furthermore, the level of *R. bromii* remained unchanged by the supplement whatever the group of mice (Supplementary Figure 7). Hence, in our study, the difference in the response of gut microbiota to SymbioIntest^®^ supplementation does not seem to be linked to *R. bromii*. Interestingly, it has recently been reported in an *in vitro* study that not only the *Ruminococcaceae* but also the *Prevotellaceae* family are both primary assimilators of RS (Herrmann et al., 2017). In our study, the gut microbiotas differed between the different donors in the *Ruminococcaceae* and *Prevotellaceae* families. The bacterial genus that mostly accounts for the differences in the *Ruminococcaceae* family is *Faecalibacterium*: the most responsive gut microbiota to SymbioIntest^®^ have the lowest level of *Faecalibacterium* and the highest level of *Prevotellaceae*; whereas the gut microbiota resistant to the supplement has the highest level of *Faecalibacterium* and the lowest level of *Prevotellaceae*. Interestingly, the gut microbiota of D3, intermediary at the level of *Faecalibacterium* with no *Prevotellaceae* detected, has a specific response to the dietary supplement. This data highlights that the interactions between the gut microbiota and RS are highly complex, and that the levels of several populations of genera involved in RS breakdown must be taken into account.

Several studies highlight that RS might contribute to positive health outcomes and this could involve a beneficial effect on the function of the large bowel (Bindels et al., 2015). Particularly, in humans, RS is fermented by the gut microbiota into SCFA, especially into butyrate (McOrist et al., 2011; Shen et al., 2017). It is well described that SCFA have a diverse range of physiological effects on the host: they are used as fuel for intestinal cells, maintain mucosal integrity and modulate intestinal inflammation (Macfarlane and Macfarlane, 2012). In the mice responding to SymbioIntest^®^ supplementation, we found that the product resulted in an increase of caecal butyrate concentration. In addition and in line with Maier et al. (2017), we also observed an increase of propionate in two out of four groups of mice (those inoculated with D2 and D3 gut microbiota).

As discussed above, organisms targeted by SymbioIntest^®^ belong to the bacterial families of *Lachnospiraceae* and *Ruminococcaceae* that are linked to the formation of butyrate (Anand et al., 2016). Impact of RS on these bacterial families of the gut microbiota has been previously described in overweight individuals (Walker et al., 2011; Salonen et al., 2014), in individuals with reduced insulin sensitivity (Maier et al., 2017) or in piglets (Umu et al., 2015). Some of these organisms have been demonstrated to have a health-promoting impact, in particular *Faecalibacterium prausnitzii* for which an anti-nociceptive (Miquel et al., 2016) or anti-inflammatory (Miquel et al., 2015) effect has been described.

Our study shows that SymbioIntest^®^ supplementation elicited a beneficial effect on the function of the large bowel in healthy adult gut microbiota by increasing caecal SCFA production especially butyrate, and by improving health-promoting taxa. However, we suggest that inter-individual differences in gut microbiota populations may result in varying abilities to utilize the supplement. As suggested by others (Healey et al., 2017; Kuntz and Gilbert, 2017), we highlight the fact that the gut microbiota is informative in predicting individualized responses to dietary intervention and developing personalized strategies.

## Ethics Statement

Experiments were performed on the Anaxem platform of the MICALIS Institute (INRA, Jouy-en-Josas, France). The Anaxem facilities are accredited by the French “Direction Départementale de la Protection des Populations (DDPP78),” accreditation number A78-322-6. All procedures involving animal experimentation were carried out according to the European guidelines for the care and use of laboratory animals under the authority of a license issued by the French Veterinary Services (authorization number 78–122 specific to CC) and were approved by the French “Ministére de l’Enseignement Supérieur et de la Recherche” (authorization number APAFIS#3441-2016010614307552).

## Author Contributions

CC and PL conceived and designed the experiments. DB, CC, VR, and CM performed the experiments. DB and CC analyzed the data. CC, PL, and AS wrote and revised the manuscript. All authors reviewed the manuscript.

## Conflict of Interest Statement

AS is an employee of the Institut für Mikroökologie, Herborn, Germany. DB had a temporary contract with Symbiopharm for 12 months. The remaining authors declare that the research was conducted in the absence of any commercial or financial relationships that could be construed as a potential conflict of interest.
