# Supplementary material for: Modulation of the Caecal Gut Microbiota of Mice by Dietary Supplement Containing Resistant Starch: Impact Is Donor-Dependent
Source: Front Microbiol. 2019 Jun 6;10:1234. doi: 10.3389/fmicb.2019.01234 (PMC6563722; doi:10.3389/fmicb.2019.01234)
Supplement: Supplementary file 1 [file Data_Sheet_1.PDF]

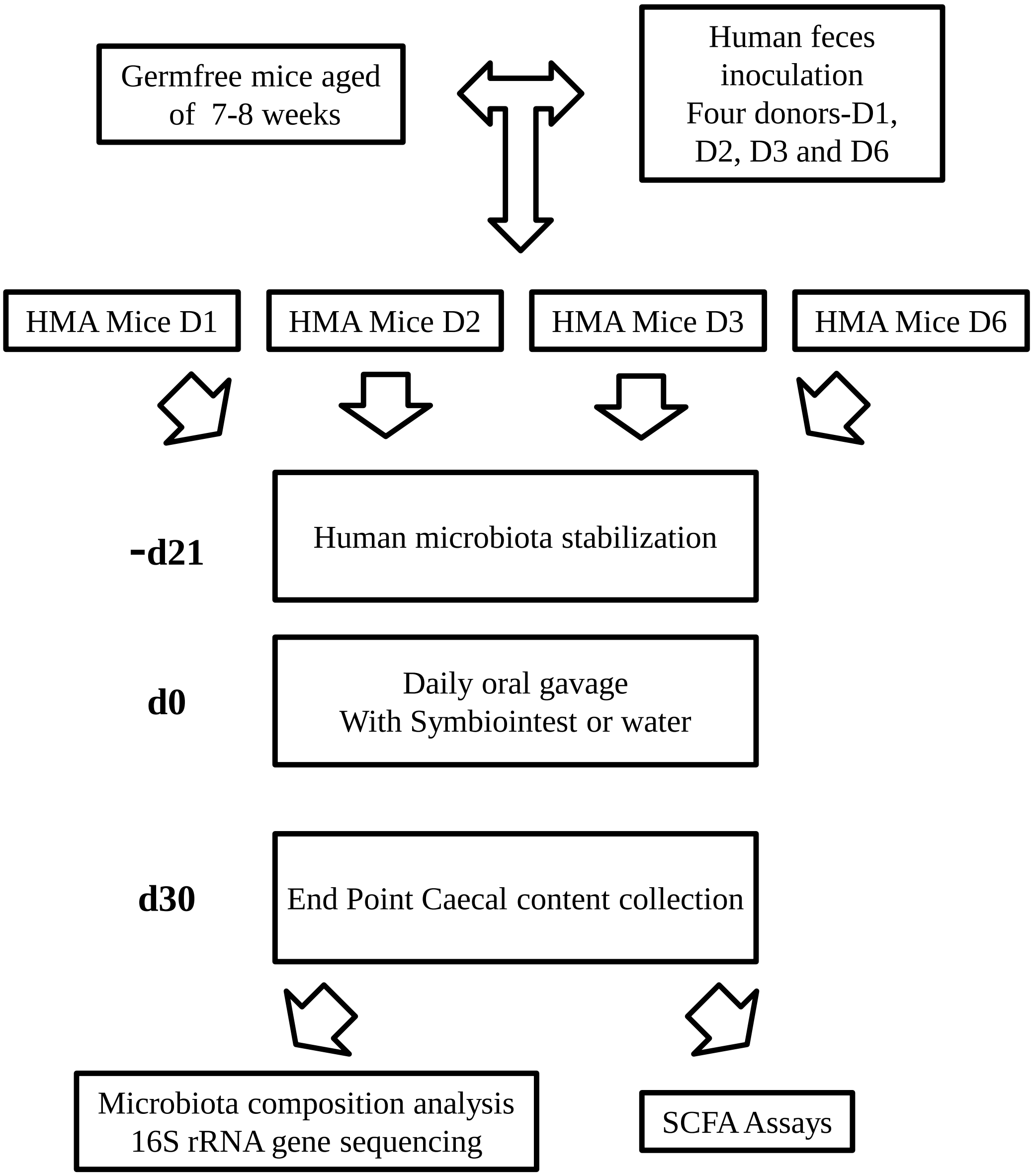

Supplemental Fig.1 : Diagram of the experimental flow .

HMA: Human Microbiota associated. SCFA: Short Chain Fatty Acid.

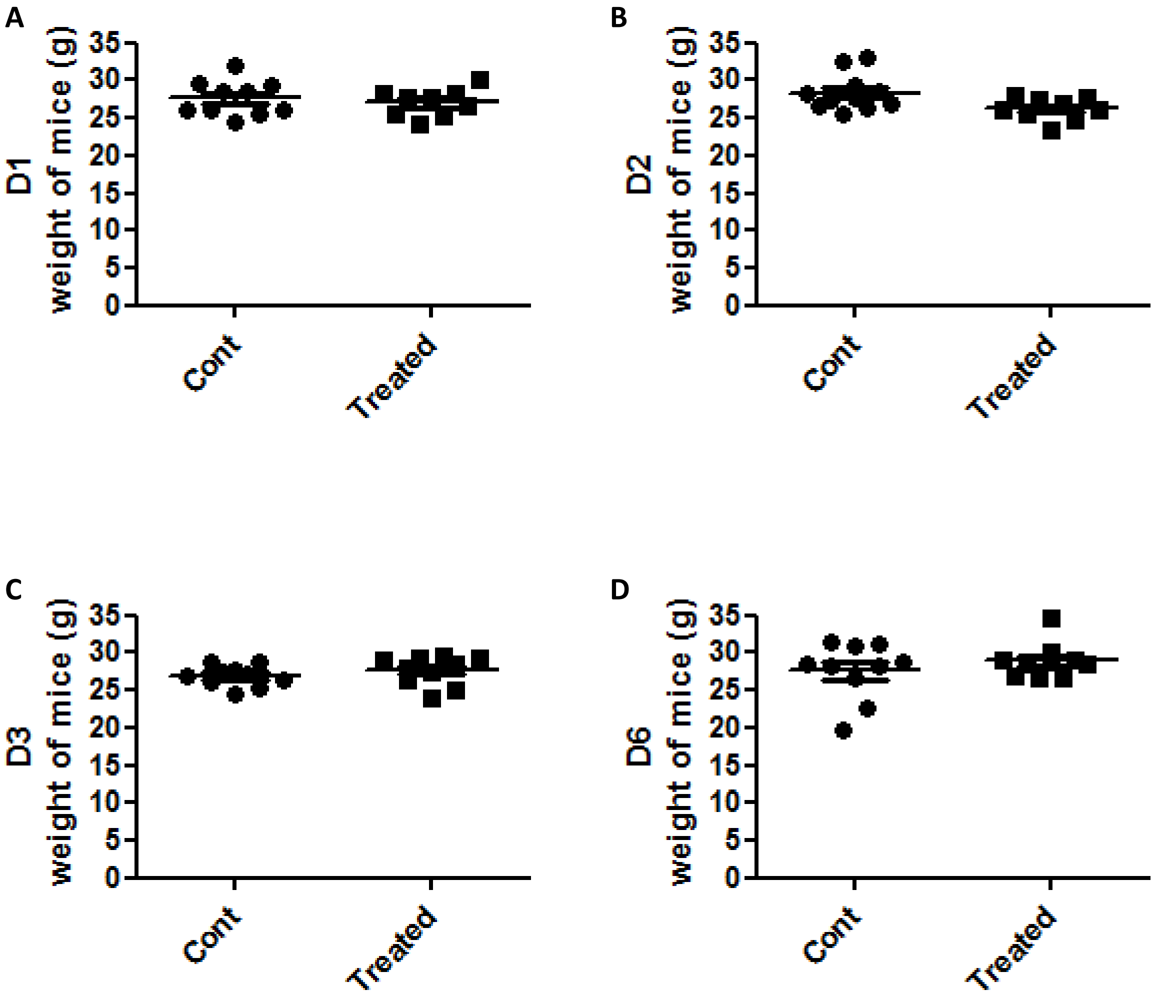

Supplemental Fig.2 :

Mice inoculated with faeces from donors D1 (A), D2 (B), D3 (C) and D6 (D) were weighed four weeks after daily supplementation with water (Cont) or SymbioIntest® (Treated) i.e. at the end of the experiment. No significant difference (non-parametric Mann-Whitney test; $p < 0.05$) was observed between the control and treated groups. Each dot represents one mouse with 9 to 10 mice analyzed per groups.

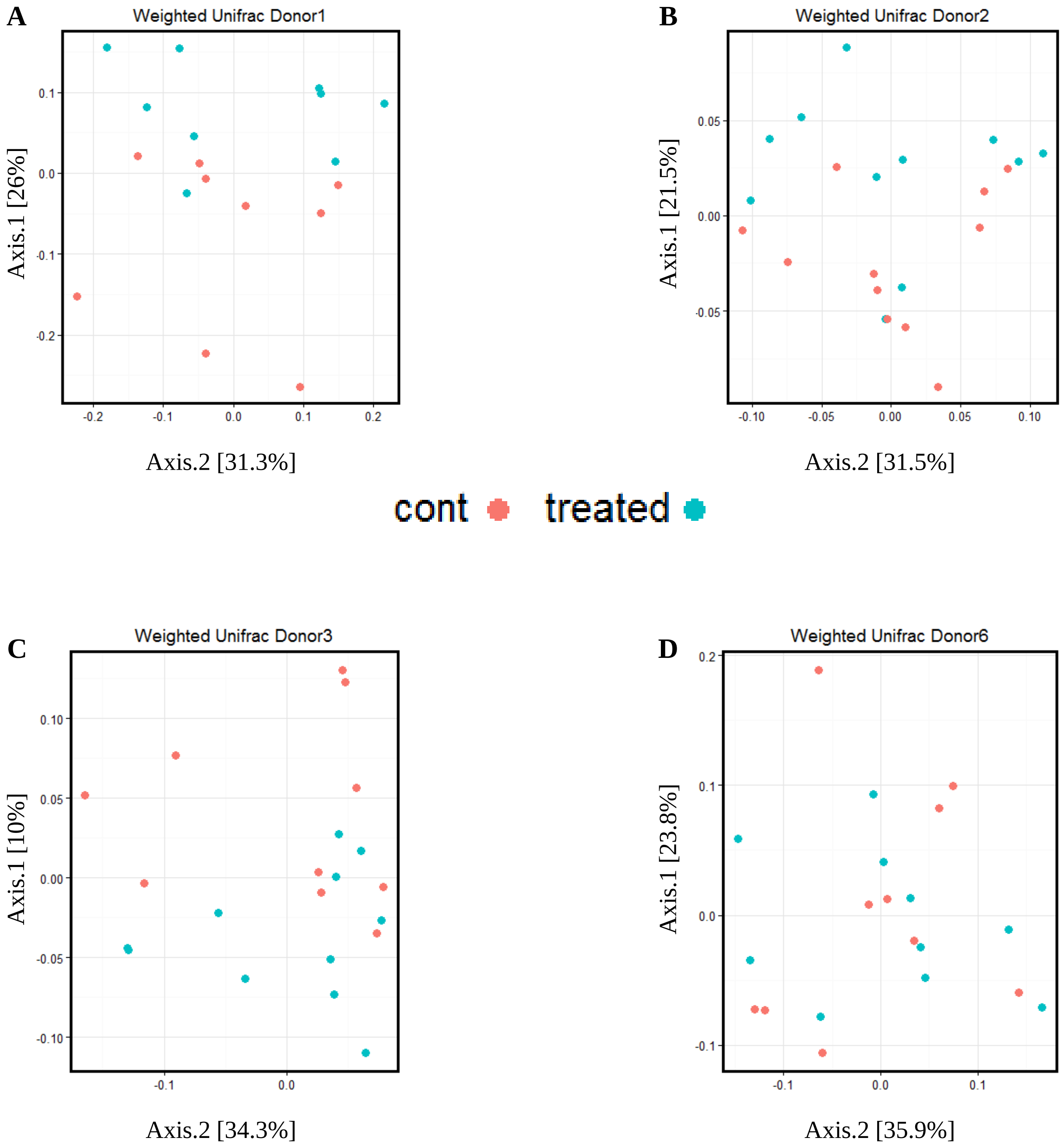

Supplemental Fig.3 :

Caecal contents were collected from mice inoculated with faeces from donor D1 (A), D2 (B), D3 (C) and donor D6 (D) after four weeks of daily supplementation with SymbioIntest® or water. 16S rDNA gene sequences were obtained and the composition analysed by Weighted Unifrac distance. Cont and treated: data obtained from water and SymbioIntest®-supplemented mice, respectively. Each dot represents one mouse with 9 to 10 mice analyzed per groups.

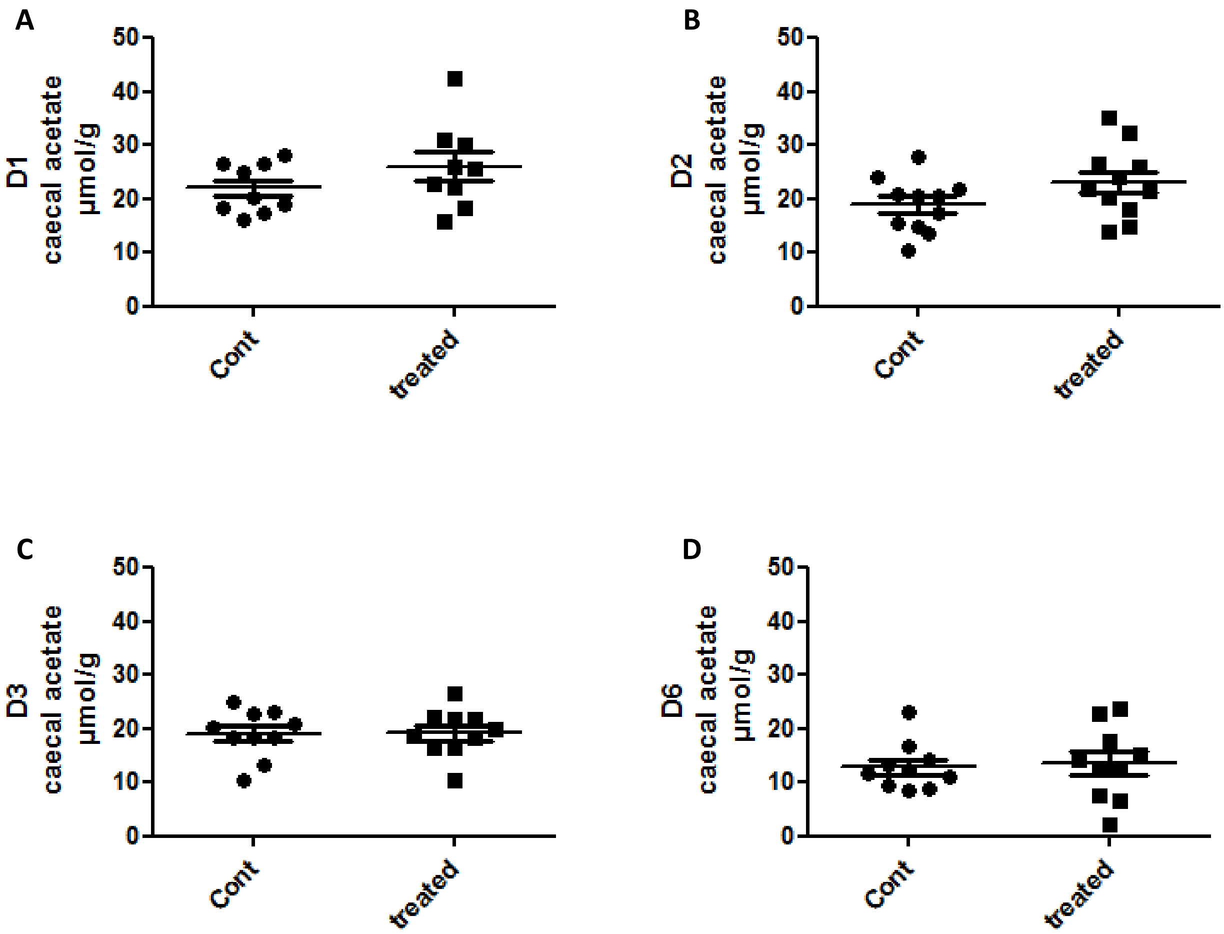

Supplemental Fig.4 :

Caecal contents were collected from mice inoculated with feces from donors D1 (A), D2 (B), D3 (C) or D6 (D) after four weeks of daily supplementation with SymbioIntest® and processed for acetate measurement as described in the methods section. Cont and treated: caecal contents obtained from control and SymbioIntest® -supplemented mice. No significant differences (non-parametric Mann-Whitney test; $p < 0.05$) were observed between the control and treated groups. Each dot represents one mouse with 9 to 10 mice analyzed per groups.

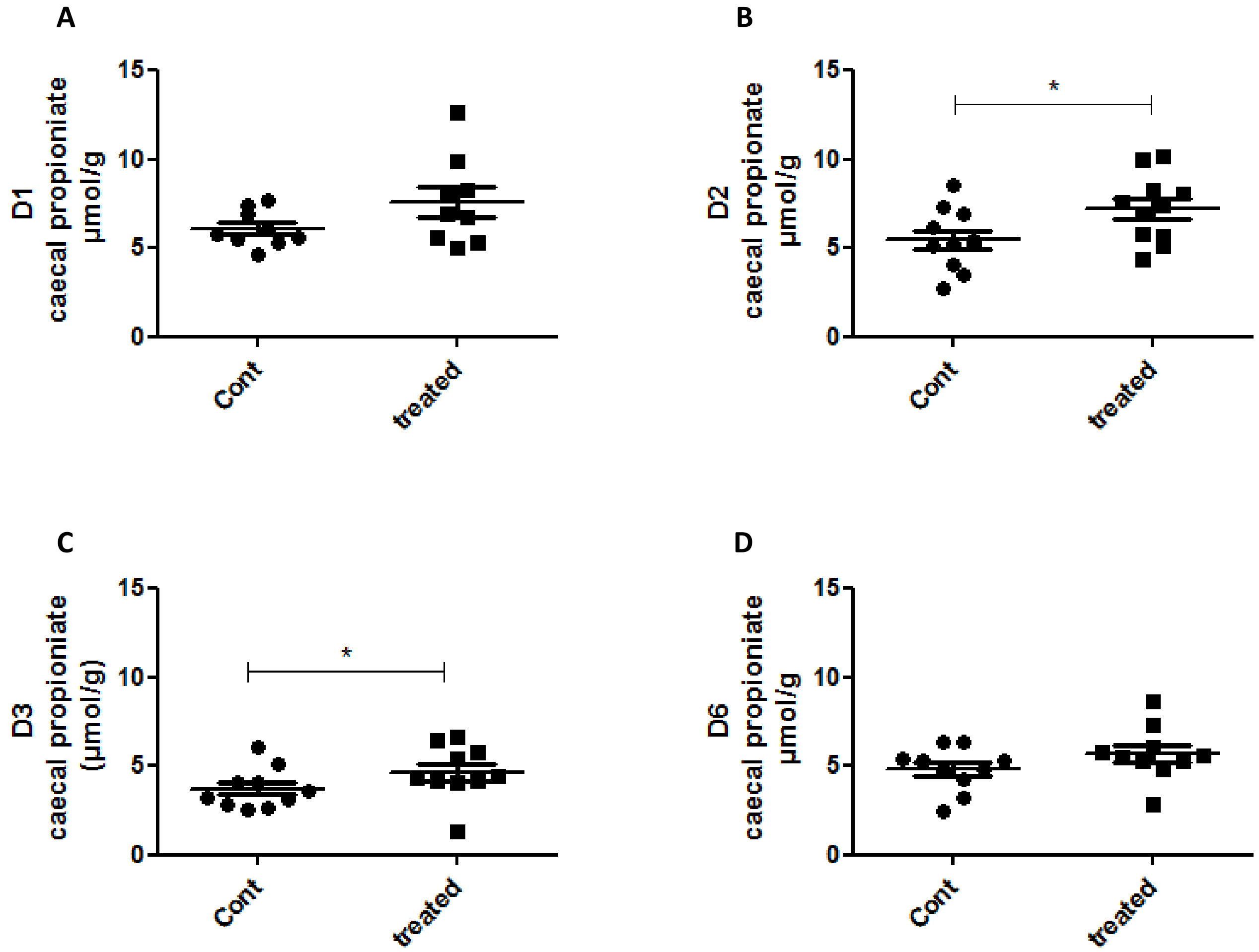

Supplemental Fig.5 :

Caecal contents were collected from mice inoculated with feces from donors D1 (A), D2 (B), D3 (C) or D6 (D) after four weeks of daily supplementation with SymbioIntest® and processed for propionate measurement as described in the methods section. Cont and treated: caecal contents obtained from control and SymbioIntest® -supplemented mice, respectively. * $p<0.05$ (non-parametric Mann-Whitney test). Each dot represents one mouse with 9 to 10 mice analyzed per groups.

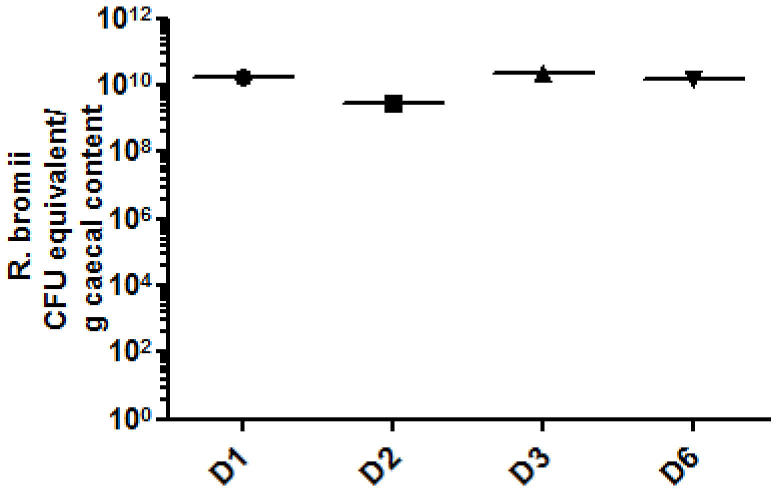

Supplemental Fig.6:
Quantification of levels of *Ruminococcus bromii* in the gut microbiota of donors D1, D2, D3, and D6 by QPCR. PCR conditions and primers used are those described in (1).

(1) Walker AW, Ince J, Duncan SH, Webster LM, Holtrop G, Ze X et al. (2011). Dominant and diet-responsive groups of bacteria within the human colonic microbiota. ISMEJ 5: 220–230

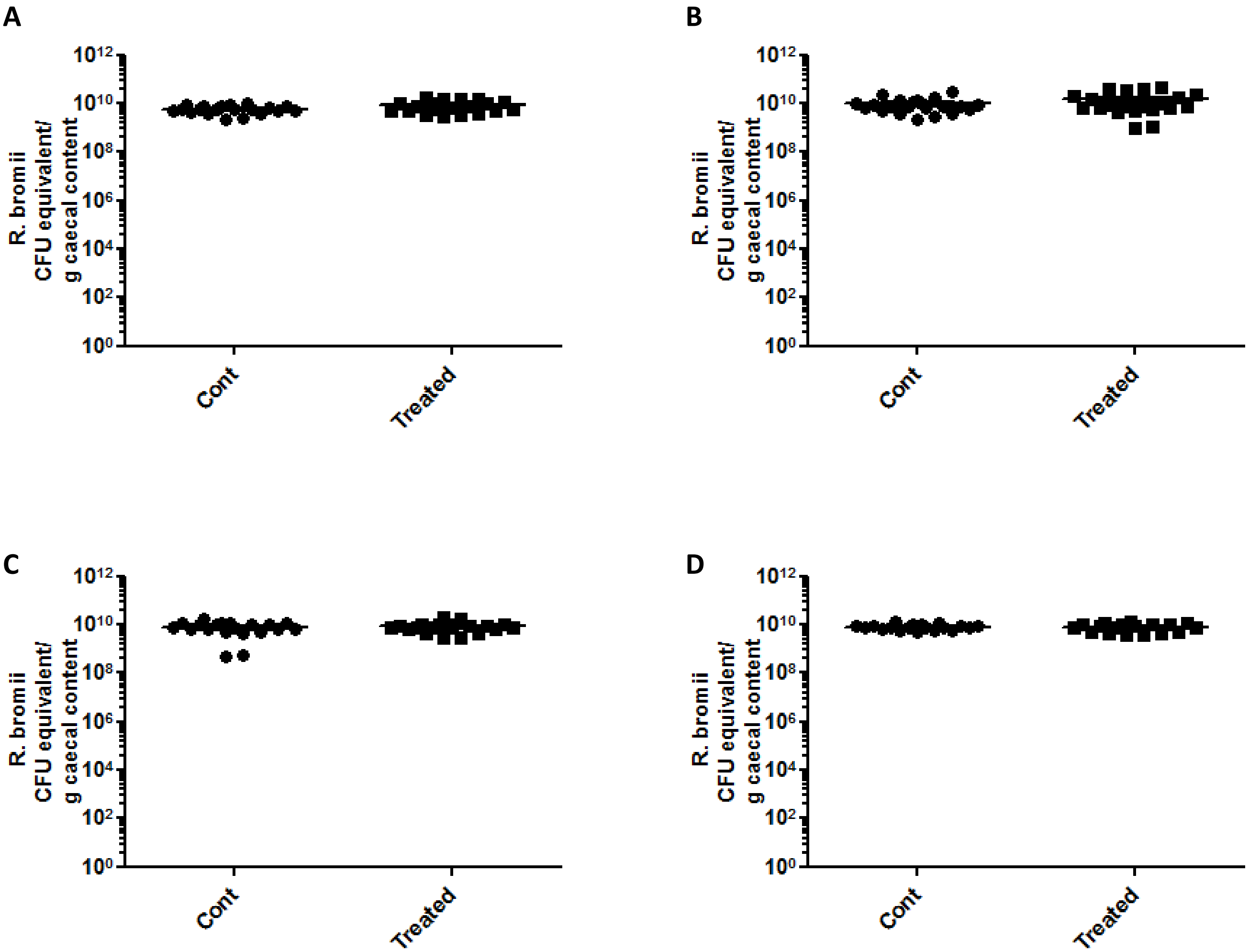

Supplemental Fig.7:

Quantification of levels of *Ruminococcus bromii* in the gut microbiota of mice inoculated with the gut microbiota of donors D1 (A), D2 (B), D3 (C), and D6 (D) by QPCR after four weeks of daily supplementation with SymbioIntest® . Cont and treated: caecal contents obtained from control and SymbioIntest® -supplemented mice. PCR conditions and primers used are those described in (1). Data is presented as mean SEM of CFU equivalent / g of faecal samples. No significant differences (non-parametric Mann-Whitney test; $p < 0.05$) were observed between the control and treated groups. Each dot represents one mouse with 9 to 10 mice analyzed per groups.

(1) Walker AW, Ince J, Duncan SH, Webster LM, Holtrop G, Ze X et al. (2011). Dominant and diet-responsive groups of bacteria within the human colonic microbiota. ISMEJ 5: 220–230

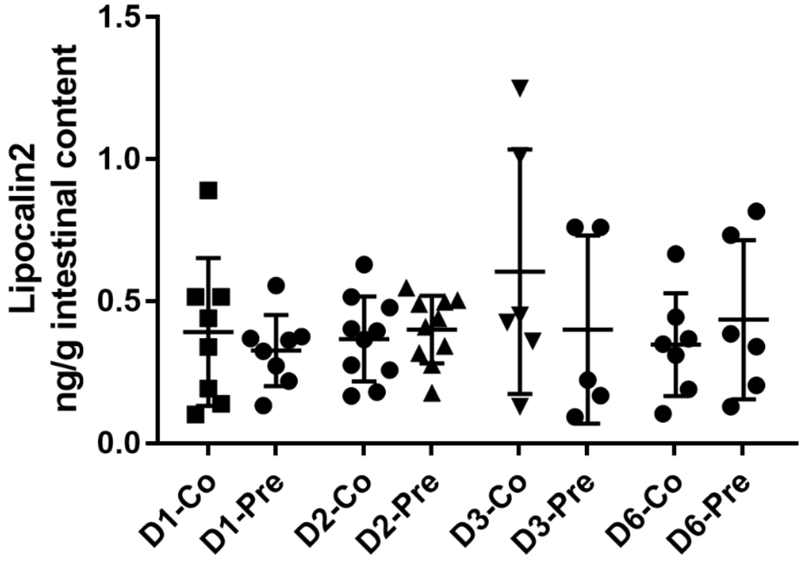

Supplemental Fig.8:

Lipocalin 2 levels in the intestinal contents of mice inoculated with feces of donors D1 , D2 , D3 , and D6 , which have received the prebiotic (Pre) or water (Co). Fecal lipocalin 2 levels were analyzed by ELISA.

No significant differences (non-parametric Mann-Whitney test; $p < 0.05$) were observed between the control and treated groups. Each dot represents one mouse with 5 to 9 mice analyzed per group.
